# Academic procrastination and suicidality: A systematic review

**DOI:** 10.1192/j.eurpsy.2025.2045

**Published:** 2025-08-26

**Authors:** I. Delgado, A. Criado, B. R. Merino, J. Muñoz

**Affiliations:** 1 Universidad Isabel I, Burgos, Spain

## Abstract

**Introduction:**

Academic procrastination is the deliberate action of postponing the completion of academic tasks that must be completed, despite the harmful effects that not completing them may entail, having a particularly high prevalence in university students. Numerous studies have analyzed the consequences of academic procrastination on mental health. Furthermore, scientific evidence has also found a high prevalence of suicide risk in youth and adolescents. Therefore, it is worth asking if academic procrastination is related to the risk of suicide and self-harming behavior in students.

To our knowledge, this is the first systematic review to analyze the relationship between academic procrastination and suicidality.

**Objectives:**

To analyze 1) the relationship between academic procrastination and suicidal tendencies, 2) whether this relationship, if it exists, is influenced by other variables.

**Methods:**

Academic Search Premier, APA PsycArticles, APA PsycInfo, PSICODOC, Psychology and Behavioral Sciences Collection, MEDLINE, E-Journals, ERIC and Scopus were searched during October 2024. An additional search was also conducted using the Google Scholar search engine. The review was carried out following the criteria of the PRISMA 2020 declaration. Observational studies that analyzed the relationship between procrastination and suicidality were included, without language or time restrictions. Single case studies or case series, studies examining procrastination in non-academic settings, and studies using qualitative methodology were excluded. Each study was narratively summarized.

**Results:**

Ninety-three studies were identified; after eliminating duplicates and those works that did not meet the eligibility criteria, four studies were included for review. These studies varied in their origin (two articles from the United States, one from Spain, one from Peru, and one from Jordan) and the secondary variables evaluated. All studies found a positive and significant relationship between suicidality and academic procrastination (with correlation coefficients ranging from 0.19 to 0.51), observing a slightly higher correlation in women compared to men. Self-control was found to mediate the relationship between procrastination and suicidality.

**Conclusions:**

Our findings suggest a strong positive relationship between academic procrastination and suicidality. However, there are still few studies that analyze this topic, so it is necessary to continue researching in this field.

**Disclosure of Interest:**

None Declared

